# Frontiers in regenerative medical materials: Comments from the participants of the 2014 China–Korea Symposium on Biomimetic and Regenerative Medical Materials

**DOI:** 10.1093/rb/rbu020

**Published:** 2015-02-09

**Authors:** Jun Ma, Insup Noh, In-Seop Lee, Shengmin Zhang

**Affiliations:** ^1^Advanced Biomaterials and Tissue Engineering Center, Huazhong University of Science and Technology, Wuhan 430074, China; ^2^Department of Chemical and Biomolecular Engineering, ^3^Convergence Institute of Biomedical Engineering and Biomaterials, Seoul National University of Science and Technology, Seoul 139-743 Korea and ^4^Institute of Natural Sciences, Yonsei University, Seoul 120-749, Korea

Based on the last 10 successful series of the China–Korea Symposium on Biomaterials and Nano-biotechnology, we initiated a new invitation-based bilateral forum for established leaders and emerging young scientists in the field, the 2014 China–Korea symposium on biomimetic and regenerative medical materials, which was held from November 26 to 28 in Wuhan, China. In the past decade, a lot of breakthrough achievements in biomedical materials and regenerative medicine have been made in our two countries. Currently, biomaterials science and engineering has been evolved into a critical stage of bioactive integration and tissue regeneration from a simple functional replacement and substitution. Biomimetic and regenerative medical materials will become main topics in the field. The aim of this symposium is to provide a platform for the frontier discussion and the bilateral cooperation between China and Korea. During the symposium, we organized a special panel discussion. The participants brainstormed a lot of new ideas on how to develop this dynamic field. The topics even involved all aspects of the relationships between National Natural Science Foundation of China (NSFC) and National Research Foundation (NRF) of Korea, Chinese Society for Biomaterials (CSBM) and Korean Society for Biomaterials (KSBM), along with some strategies to improve such partnerships among institution, professional society and industrial community.

Herein, we present commentaries from some active participants that reflect their perspectives on future trend of the field.

## Jin Ho Lee


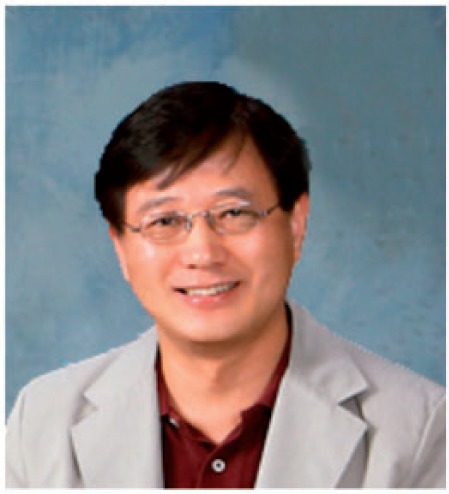


Jin Ho Lee, graduated Department of Chemical Engineering, Hanyang University, Korea with B.S. degree in 1979, Department of Chemical Engineering, Seoul National University, Korea with M. S. degree in 1981, and Department of Materials Science and Engineering, University of Utah, USA with Ph.D. degree in 1988. He worked at Biomaterials Laboratory, Korea Research Institute of Chemical Technology (KRICT) as a senior research scientist (1988–93). Since 1993, he is a professor in the Department of Advanced Materials, Hannam University, Korea. He was the Editor-in-Chiefs of ‘Biomaterials Research’ (Korean Society for Biomaterials, 2003–7) and ‘J of Korean Wound Care Soc’ (2007–10). He served as Secretary General of seventh Asian Symposium on Biomedical Materials (ASBM-7, 2006) and Scientific Program Chair of second TERMIS World Congress (2009). He also served as a President of Korean Tissue Engineering and Regenerative Medicine Society (KTERMS) (2012) and served as Conference Chair in TERMIS-AP Meeting this year (September, 2014). His recent research areas include stem cells/biocompatible polymer hybrid materials for the treatments of urinary/fecal incontinence, bioactive agents (growth factors, genes and stem cells)-incorporated nonporous/porous matrices/hydrogels for tissue regenerations such as cartilage, bone, muscle, trachea and nerve, guided tissue regenerations, and anti-tissue adhesion barrier membranes or hydrogels.

***Comment:**** Biomaterials have been one of the main parts of the tissue engineering and regenerative medicine, over the past 30 years. In the 2014 China–Korea Symposium held in Wuhan, many topics presented were also related with biomaterials-oriented tissue engineering. In spite of accumulated research and funding worldwide, there are still few tissue engineering products commercialized. We should focus more to reduce the gap between basic research and clinically applicable/commercializable outputs. In my opinion, one of those approaches to be focused in the future will be the development of bioactive matrices (including scaffolds, membranes and* in situ *injectable hydrogels) inducing self-tissue regeneration without involving cells (*in vivo *homing or guiding cells or growth factors).*

## Gang Li


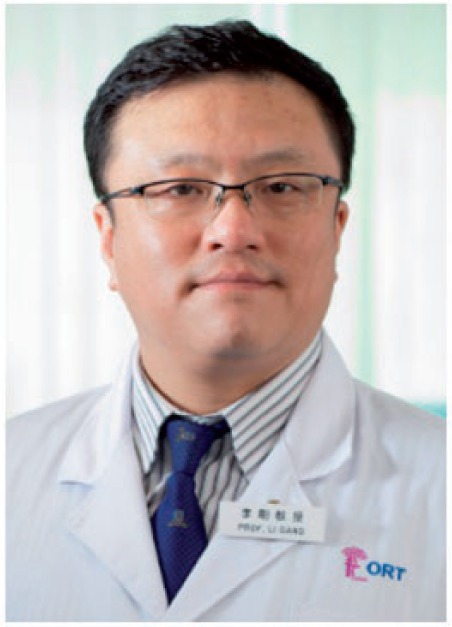


Prof. Li Gang graduated from University of Oxford Medical School (1997) with a D.Phil. degree on studies of biological mechanisms of distraction osteogenesis. After post-doctoral training at the MRC Bone Research Laboratory in the University of Oxford, he took up a lectureship (1998), Senior Lectureship (2001) and Readership (2004) in the School of Medicine, Queen’s University Belfast, UK. Dr Li is currently a Professor at the Department of Orthopaedics and Traumatology, The Chinese University of Hong Kong (2009-now). His main research interests are on biological mechanisms of distraction osteogenesis, fracture healing, musculoskeletal tissue regeneration with emphasis on stem cell biology and clinical applications. He has published more than 120 peer-reviewed SCI articles, 15 book chapters, edited 3 books on tissue engineering, distraction histogenesis, leg-lengthening and Ilizarov techniques. Prof. Li serves as associate editor of Journal of Orthopaedic Translation; member of editorial board of Calcified Tissue International (2004-now). He served as Honorary Treasurer of British Orthopaedic Research Society (2004–6), Member of Programme Committee of American Orthopaedic Research Society (2006–7) and currently is the general secretary of International Chinese Musculoskeletal Research Society (ICMRS). Prof. Li is a council member of Chinese Orthopaedic Research Society, Chinese Medical Association; council member of Tissue Engineering and Regenerative Medicine Society, Chinese Association of Biomedical Engineering.

***Comment:*** *Tissue engineering concept has been widely accepted but with limited practical/application guidance. Many surgeons and physicians wrongly think that tissue engineering must involve cells, biomaterials or bioactive factors. In fact, the human body is a big bioreactor and contains all the necessary ingredients for tissue engineering! For instance, distraction histogenesis will lead to regeneration of all sorts of musculoskeletal tissues under simple mechanical (tensile force) stimulation in a controlled manner. The distraction histogenesis technique is a living proof that human body has the regenerative potentials even at an old age, but the regenerative potentials need to be activated or promoted by physical, chemical or biological means. I believe that the future tissue engineering shall focus on developing intelligent biomaterials that could modulating the tissue repair process or mobilizing/activating the endogenous precursor cells. The intelligent biomaterial shall have all the other essential requirements as biomaterial, but with added self-assembly/remodeling and slow release of bioactive molecules. In addition, we also need to develop functional assessment standards for clinical outcome, as the golden standard for assessing any novel biomaterials. In essence, the tissue engineering approaches have to be simple and effective, with firm clinical outcome improvement, to enable their wider application and promotion.*

## Xingyu Jiang


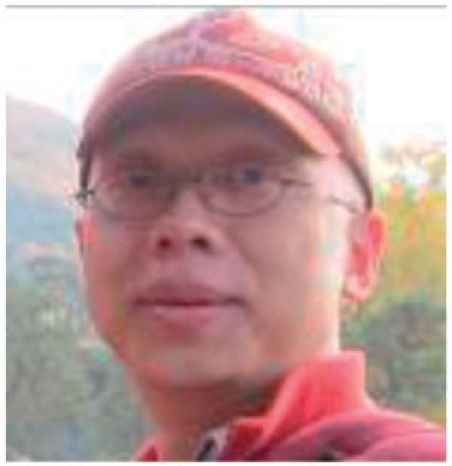


Xingyu Jiang is a Professor at the National Center for NanoScience and Technology (NCNST), China. He obtained his B.S. at the University of Chicago (1999) and Ph.D. at Harvard University. He was awarded the ‘Hundred Talents Plan’ of the Chinese Academy of Sciences, the Scopus Young Researcher Gold Award, the Chinese Chemical Society Prize for Young Chemist, The National Science Foundation of China’s ‘Distinguished Young Scholars’ Award. He has published over 140 peer-reviewed papers. He is an associate editor of Nanoscale (Royal Society of Chemistry, UK).

***Comment:**** Regenerative biomaterials will have fascinating developments in the next decade for many reasons. One of the reasons will be the emergence of new materials and new ways of fabricating known materials. Another reason is that new ways of modulating the behaviors and fates of cells will usher in capabilities that can only be imagined before. A third reason is that new models, both* in vitro *and* in vivo*, will help researchers and clinicians more effectively evaluate new biomaterials for translational work. I am very excited to be able to witness these developments.*

## Jong-Chul Park


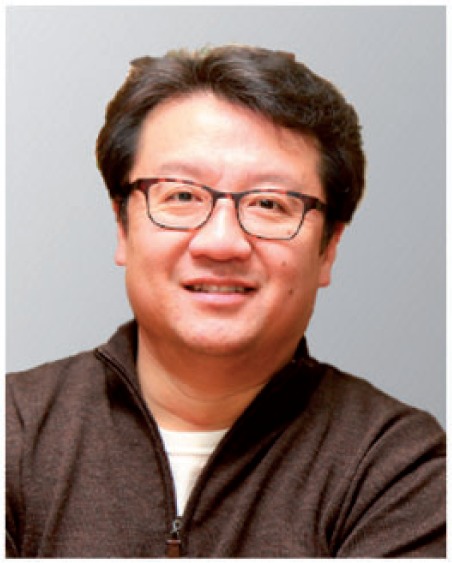


Professor and Head of the Department of Medical Engineering at the Yonsei University College of Medicine, Director of Yonsei Medical Technology and Quality Evaluation Center (MTEC) at the Severance Hospital. He received B.S. in Biochemistry from Yonsei University, Korea, in 1987 and M.S. in Public Health from Seoul National University, Korea, in 1990. He obtained Ph.D. in Biotechnology and Life Science from Tokyo University of Agriculture & Technology, Japan in 1995. He has authored or co-authored over 200 publications. His research interests focus on enhancing the properties of biomaterials and scaffolds such as biocompatibility and interactions between cells and scaffold/biomaterials for regeneration of tissue.

***Comment:*** *In modern medicine, the advance of biomaterials plays a prominent role with the great challenges in the treatment of patients. Biomaterials are used as physical replacement of hard or soft tissues which are damaged or destroyed by some pathological process or accidents. In this respect, biomaterials in clinical medicine are applied to the medical devices which have the potential risk of harm in the various fields, such as orthopedic, cardiovascular or circulatory system, ophthalmic, dental, drug delivery systems and so on. The development of biomaterials requires a great deal of time and costs for the approval to be regulated as medical devices in the clinic. In biomedical research, biomaterials should be developed with suitable design for its primary intended purposes and demonstrated the biological activity of device in cell culture and relevant animal models according as basic research and translational research without significant side effects or safety issues. Furthermore, the clinical trials should be approved by the relevant regulatory bodies before they are used to be therapeutic applications to treat patients. In the past decade, biomaterials have been developed by multidisciplinary research to regenerate damaged tissue in the fields ranging from cell biology to materials science to chemistry to clinical medicine. In medicine, biomaterials to regenerate damaged tissue have always been employed by surgeons. That is why research must be approached by demand of medical doctors as inventors for development of new biomaterials. Consequently, future study should be reflected clinician’s unmet needs and understand of incredibly complex human body with careful consideration of the challenges moving from the laboratory bench to the patient’s bedside to make a large clinical impact.*

## Deling Kong


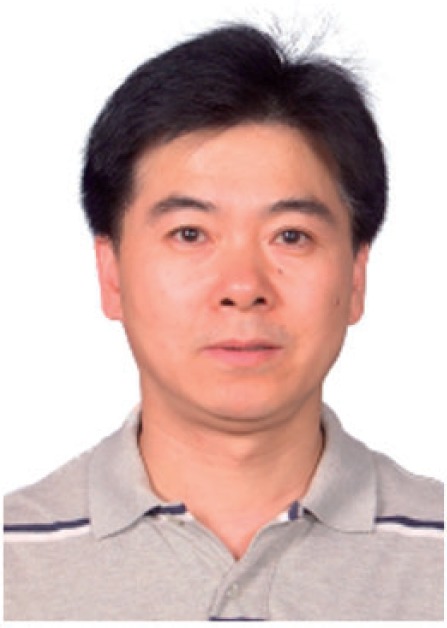


Professor of biochemistry and molecular biology at Nankai University, Director of the Key Laboratory of Bioactive Materials of Ministry of Education at Nankai University, vice Director of Institute of Biomedical Engineering at Chinese Academy of Science & Peking Union Medical College. He obtained his Ph.D. in polymer chemistry from Nankai University in 1997, and completed his postdoctoral training at Brigham Women’s Hospital in Harvard Medical School in 2003. His research focuses on cardiovascular tissue engineering, including small diameter vascular grafts, bioactive hydrogel and molecular probes for stem cell therapy. He has co-authored over 200 research articles, and is the owner of more than 10 Chinese patents. He received the NSFC Outstanding Youth Fund in 2007. He won the second class National Scientific Technology Progress Award of China in 2009, and the second class Natural Science Award of Tianjin in 2010.

***Comment:*** *Traditionally, the interaction between biomaterials and the immune system has been studied mainly in the context of two separate domains of practice-biocompatibility evaluation of implants and formulation of vaccines. In recent years, progress in basic immunology and immunotherapy has produced new knowledge and new insight on how the immune system works at the cellular and molecular levels. Mechanism-driven molecular engineering of biomaterials is now being attempted toward active modulation of the immune system, rather than simply being passive and inert, giving rise to new and more effective vaccine carriers, gene and drug delivery systems, and functional scaffolds for rapid tissue repair and regeneration. Such effort will surely have profound positive impact on the prevention and treatment of human diseases.*

*In light of the recent development in this important interdisciplinary field, Dr Chun Wang (University of Minnesota) and me coined the term ‘immunobiomaterials’ to define the revolutionary paradigm-shift toward immunologically driven engineering of biomaterials, and we organized the first International Symposium on Immunobiomaterials in September 20–22, 2014 in Tianjin. The Symposium has brought together some of the leading experts worldwide to introduce state-of-the-art of engineering biomaterials for modulating the immune system and to discuss future possibilities and challenges.*

## Yu-Shik Hwang


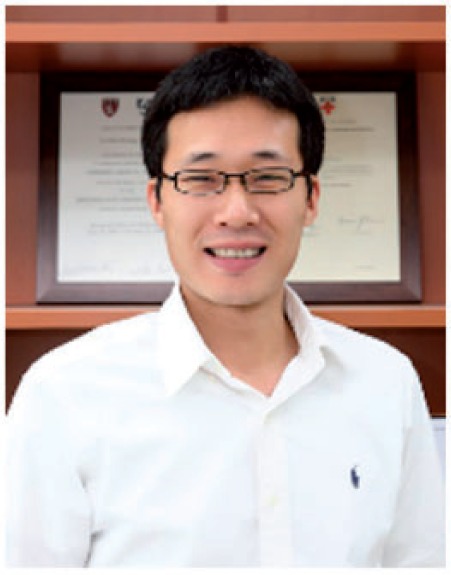


Assistant Professor of Maxillofacial Biomedical Engineering at School of Dentistry of Kyung Hee University. He obtained B.Sc in Biochemistry and M.Sc in Biomedical Engineering from Yonsei University and Ph.D. in Chemical Engineering from Imperial College London, and completed his postdoctoral training in the field of BioMEMs at Harvard-MIT HST in Boston. He has authored over 50 original and review articles. Currently, his laboratory focuses on natural polymer-based smart biomaterials and integrated bioprocess to develop micro tissues for tissue engineering and regenerative medicine.

***Comment:*** *Over the past decades, there have been remarkable advances in biomaterials development, and extensive research on stem cell has been done. The coordination of the fields of biomaterials and stem cell biology has changed insight into the needs of smart biomaterials to control stem cell’s growth and lineage-specific differentiation. For translational research from bench to clinics, clinical trials to regenerate tissues and organs has been designed and animal study has been done actively by the transplantation of newly developed smart biomaterials or stem cell or composites. Many researches have shown the good outcome to regenerate tissue effectively via biomaterial-guided stem cell growth and desired tissue formation through host or transplanted stem cell differentiation. However, few recent studies have shown the precise mechanism and underlying biology of how smart biomaterials control stem cell growth and differentiation. A good understanding of the underlying biology of cellular interaction of biomaterials will be helpful to improve and design a more reliable and promising technology to develop tissue engineered tissue or to regenerate tissues. Regarding microenvironment around cells and tissue* in vivo *is very complicated, more aspects about cell-to-biomaterial interaction regarding* in situ *cell-to-ECM, cell-to-cell and cell-to-soluble factors interaction need to be considered. Consequently, future studies to develop smart biomaterials should suggest precise mechanisms of their effects together with the potent clinical applications for the therapeutic promise of tissue engineering.*

## Xin Zhang


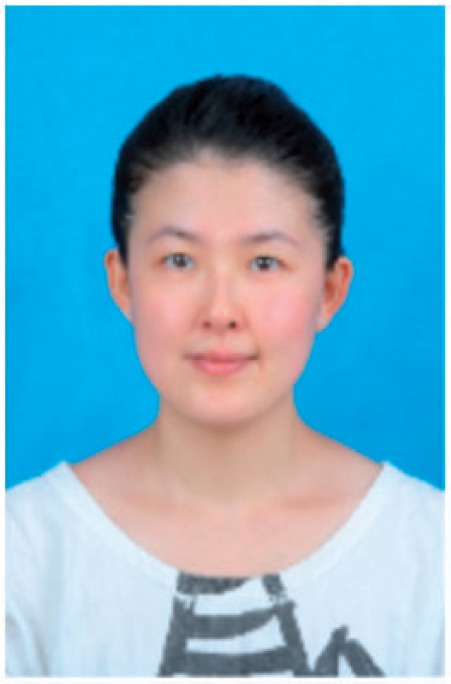


Dr Xin Zhang received her Bachelor degree and Master degree, respectively in Macromolecular Materials and Biomedical Engineering at Tianjin University, and obtained her Ph.D. at Strasbourg University. In the same year, she was invited to be an assistant researcher in the French National Institute of Health and Medical Research (INSERM). In 2010, she has become a professor in Institute of Process Engineering, Chinese Academy of Sciences. Currently, her research focuses on designing and preparing new drug delivery carrier system; developing a system for the early diagnosis of malignant tumor; the development and application of tumor vaccine adjuvants.

***Comment:*** *Regenerative medicine is an amalgamation of many disciplines, principles and concepts, which is often composed by biological part (living human cells) and nonbiological part (biomaterials as scaffold or matrix). The nonbiological scaffold offer cells a living place for proliferation, and the cell provide biological functionality to restore, maintain or enhance tissues and hence organ functions. However, the combination of living cells and materials seem not so successful in long-term clinical application, because these materials show worse biological and mechanical functionality compared to natural tissues. In some extreme visions, the living human cells are the central focus of regenerative medicine, and regenerative medicine should be toward emulating nature’s capacity, with minimum of unnatural materials involved. However, in most cases, the artificial scaffold is necessary in which it is hard to see cell alone regenerative medicine will be successful. Instead of excluding artificial materials form the system of regenerative medicine, it is a better idea to achieve the best blend of these two parts by making these artificial materials look like more ‘natural’. Thanks to the advent of nanotechnology, it opens a new window of opportunity to design and fabricate novel biomimetic materials which blend living cells and materials in natural and harmonious way. These synthetic materials resemble biological extracellular matrix, which interact with cells and control cell function at the signal-transduction level to control the processes of tissue regeneration effectively. All the materials obey the rule ‘inspired by nature’, their duties are to provide cell the most natural and comfortable environment to max the therapy effect. Such materials might eventually replace the more traditional choices, and the future of regenerative medicine materials belongs to the bio-inspired biomaterials.*

## Sebyung Kang


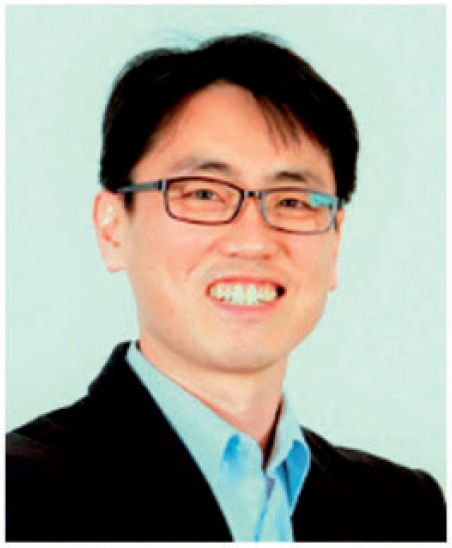


Dr Sebyung Kang is an Associate Professor of Department of Biological Sciences at Ulsan National Institute of Science and Technology (UNIST). He obtained his B.S. and M.S. degrees in Microbial Engineering at Konkuk University in Korea and Ph.D. degree in Biochemistry and Molecular Genetics at the University of Alabama at Birmingham in USA. He further performed his postdoctoral studies at the University of Alabama at Birmingham and Montana State University. He has published over 40 original research and review articles and book chapters. His research interests include nanotechnology, biomolecule-based smart drug/diagnostics delivery and biomacromolecule engineering for biomedical application.

***Comment:*** *The ideal goal of regenerative medicine is the* in vivo *or* in vitro *generation of a functional organ with a scaffold made out of synthetic or natural materials that have been loaded with living cells. Revolutionary nanotechnology allows a precise control of the interaction and localization of biomolecules, nanomaterials and cells even at the single-molecule level. Nanoscale changes in the dynamic extracellular matrix can significantly alter the cell behavior and vice versa. Biomimetic nanopatterns alone can direct the differentiation of stem cells by design without involvement of exogenous soluble biochemical factors mimicking the dynamic extracellular matrix. This directed control of cellular behavior using nano-scaled tools is one demonstrative example of significant applications of nanoengineering in regenerative medicine. Nanomaterials used in biomedical applications include nanoparticles as delivery vehicles for cargo molecules, such as drugs, growth factors, and nucleic acids, nanofibers and nano/mesoporous scaffolds for tissue and bone regeneration, nanocomposites for surface modifications of implantable materials or biosensors. The combination of these elements within tissue engineering is an excellent example of the great potential of nanotechnology applied to regenerative medicine. Nanotechnology is contributing more and more to the development of biomimetic, intelligent biomaterials, which are designed to actively respond to changes in their immediate environment and stimulate specific regenerative events at the molecular level in order to generate healthy tissues. However, the concern about the safety of nanomaterials used in regenerative medicine for human health should be thoroughly investigated.*

## Xudong Li


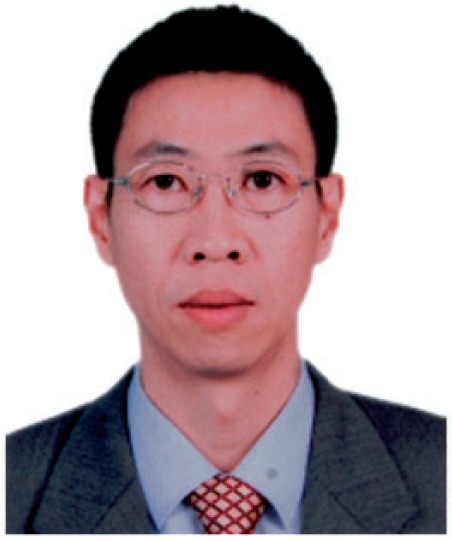


Xudong Li is a professor at the National Engineering Research Center for Biomaterials and the Engineering Research Center in Biomaterials of Sichuan University, China. He obtained his BSc (solid state physics) at Sichuan University in 1983, began his biomaterials study in 1988 and received his Ph.D. at Queen Mary, University of London. His current research interests include developing novel biomaterials for tissue repair and regeneration, preparing smart micro-/nano-carriers for guest delivery, and biomimetic mineralization.

***Comment:*** *It was a special pleasure to participate in the 2014 China–Korea Symposium which was an absolute success thanks to the elaborate organizing contributions. This symposium provided a forum for colleagues from both China and Korea to exchange their latest research achievements covering almost every element of tissue engineering and regenerative medicine and spearheading the future development of biomimetic and regenerative medical materials. All the lectures and the subsequent discussions were very exciting, informative and instructive, and would undoubtedly inspire in-depth academic exploration through the enhanced bilateral collaboration. I am looking forward to the next success of this annual symposium in Korea.*

## Jae-Young Lee


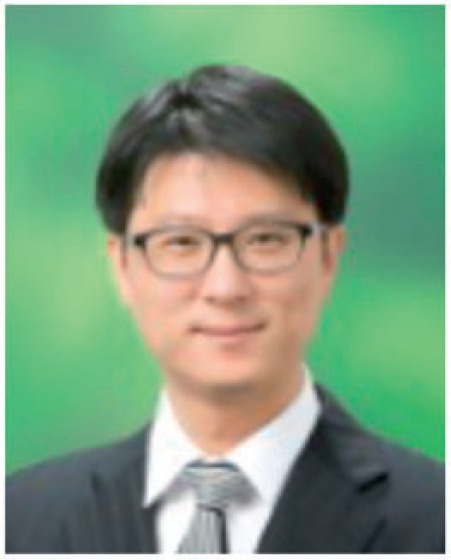


Assistant Professor of School of Materials Science and Engineering, Gwangju Institute of Science and Technology (GIST), Gwangju, Republic of Korea. Dr Lee received B.S. and M.S. degrees in Chemical Technology, Seoul National University in 1997 and 1999. He worked as an associate research manager in LG Life Science Ltd from 1999 to 2005. He received his Ph.D. from The University of Texas at Austin in 2010. He studied his postdoctoral research in University of California Berkeley with an American Heart Association (AHA) postdoctoral fellowship. He joined GIST in 2012. His research is in the field of biomaterials, which include biomimetic material design, electrically active biomaterials, plasmonic nanomaterials and stem cell applications.

***Comment:*** *Biomimetic materials have gained great attentions for the past decades in designing and employing functional biomaterials for fundamental studies on cell biology as well as therapeutic applications. Microenvironments of cells and native tissues in the body consist of numerous compounds, which determine cells/tissue fates, such as proliferation, migration, differentiation and death. Accordingly, profound efforts have been made to construct ‘artificial microenvironments’ exhibiting important characters of native cellular environments. Such key factors have been identified and can be classified as ‘biological’, ‘chemical’, ‘mechanical’, ‘topographical’ and ‘electrical’ cues. For example, recent studies have highlighted the importance of nonbiochemical characteristics of biomaterials especially with stem cell biology. Importantly, systematic studies on nonbiochemical signals delivered by biomaterials, their effects on cell behaviors and clear mechanisms are still highly required as future studies. Although individual cues have been extensively studied and employed in both* in vitro *and* in vivo *systems, advanced biomimetic materials that can simultaneously deliver multiple cues with investigation of their effects on cells/tissues will be greatly desired to better mimic cellular environments with better effectiveness as future studies. In addition, considering that the biological systems are dynamic and changes according to the conditions, it will be essential to study and develop smart biomimetic materials that present different signals with different activities in the match of time. Altogether, biomimetic materials, as artificial microenvironments, will allow us to find right paths to treat diseases and injuries.*

## Bin Li


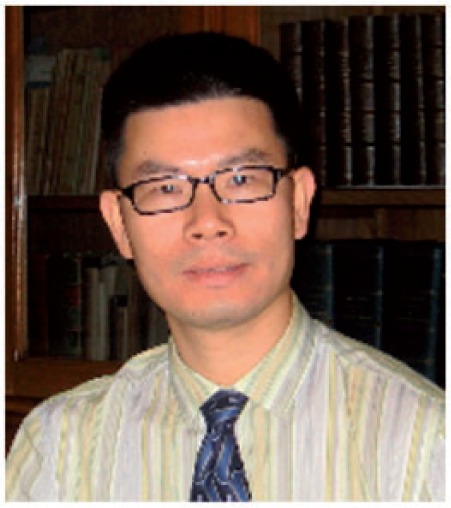


Professor Bin Li received the bachelor and Ph.D. degrees from Tsinghua University in 1996 and 2001, respectively. He then worked at the Institute of Materials Research and Engineering, Singapore, from 2001 to 2004. After that he pursued postdoctoral training at University of Pittsburgh School of Medicine in USA from 2005 to 2009. He also completed two short-term trainings as a visiting research scientist at Carnegie Mellon University in 2004 and Harvard University in 2009, respectively. He joined Soochow University as a Specially Appointed Professor in 2009. He is now the principle investigator and director of the Biomaterials and Cell Mechanics Laboratory (BCML) of Orthopedic Institute, Soochow University. He currently serves as the Member-at-Large of the Board of Directors and a member of the China Development Committee of International Chinese Musculoskeletal Research Society (ICMRS). He is also a fellow of Chinese Orthopaedic Research Society (CORS), Chinese Association of Orthopaedic Surgeons (CAOS) and Chinese Association of Rehabilitation Medicine (CARM). He serves as an editorial board member of five academic journals. He has contributed to over 70 publications. His research interests include biomaterials for bone and cartilage repair, stem cells and tissue engineering, smart molecular recognition and controlled release, surface modification and functionalization, cellular biomechanics and mechanobiology.

***Comment:*** *In tissue engineering and regenerative medicine (TERM), a fundamental role of the biodegradable scaffold is to provide a temporary housing for cells to grow and then function. I envision three categories of scaffolds will show significant impact to the progress of TERM. The first category is the scaffolds that approximate the matrix of native tissue from molecular, structural and mechanical aspects. Examples of such scaffolds include decellularized tissue/organ, cell-derived matrix and certain synthetic polymers. The second category is the scaffolds that can achieve long-term release of bioactive molecules, preferentially from nonanimal origins, in a temporally and spatially controllable manner. Orchestrating the different patterns of needs for bioactive factors at different time and sites during the whole course of tissue regeneration, while challenging, is highly desired as it recapitulates the native tissue development process. The third category is the scaffolds that possess molecular recognition capability toward specific cell surface markers or bioactive factors. By recognizing stem cells or capturing growth factors* in vivo *through the specific interaction between the recognition sites and their ligands, such scaffolds function to recruit therapeutic stem cells or enrich highly efficient endogenous growth factors, and therefore effectively promote tissue regeneration. All these scaffolds, by activating or promoting the regeneration capability of the body itself upon* in vivo *implantation, will find numerous applications.*

